# The Effect of Cu Content on the Microstructure and Properties of the Wire Arc Additive Manufacturing Al-Cu Alloy

**DOI:** 10.3390/ma16072694

**Published:** 2023-03-28

**Authors:** Lingling Ren, Zhenbiao Wang, Shuai Wang, Chengde Li, Wei Wang, Zhu Ming, Yuchun Zhai

**Affiliations:** 1 Inner Mongolia Metal Material Research Institute, Baotou 014000, China; 2 North East Industrial Materials & Metallurgy Co., Ltd., Fushun 113000, China; 3 School of Materials and Metallurgy, Northeastern University, Shenyang 110819, China

**Keywords:** WAAM, Al-Cu alloy, Cu content, microstructure, mechanical properties

## Abstract

Al-Cu alloy has broad application prospects in the field of aerospace due to its excellent performance. In this paper, deposits with different Cu contents were prepared by the wire arc additive manufacturing (WAAM) process, and the effects of Cu content on the microstructure and mechanical properties were investigated. The microstructure of Al-Cu alloy was investigated by metallography, scanning electron microscope (SEM), energy-dispersive spectrometer (EDS), and transmission electron microscope (TEM). The results show that both the number and size of the precipitated θ phases (Al_2_Cu) in the as-deposited material increase with the increase of Cu content. After the T4 treatment, the solid solution amount of Cu in the matrix showed a trend of first increasing and then remaining stable. When the content of Cu was greater than 5.65%, as the Cu content increased, the number and size of the remaining θ phases both increased. In the peak ageing state, the amount of precipitated θ’ phase showed a trend of increasing and then remaining stable. After the T6 treatment, the mechanical properties showed a trend of first increasing and then decreasing with the increase of the content of Cu. When the Cu content was 5.65%, the deposit achieved the best mechanical properties, and the anisotropy of the mechanical properties disappeared. The tensile strength, yield strength, and elongation reached 538 MPa, 478 MPa, and 10.5%, respectively. When the content of Cu was greater than 5.65%, the anisotropy of mechanical properties was obvious, and the fracture mode of the vertical specimen changed from ductile fracture to brittle fracture.

## 1. Introduction

Al-Cu alloy has a high specific strength and heat resistance and is widely used in aviation, aerospace, the military industry, automobile manufacturing, and other fields [1,2]. The three traditional forming methods of Al-Cu alloy are casting, forging, and welding. However, due to the wide range of crystallization temperatures of Al-Cu alloys, casting products are prone to defects such as hot cracking [3], segregation [4], and shrinkage porosity [5], resulting in low yield. The low material utilization rate and long production cycle of the forging process have also been criticized. The welding process experiences difficulty in matching the equal strength of the base metal and the weld. These all limit the application scope of Al-Cu alloy.

The characteristics brought by the WAAM process are a short production cycle [6], unlimited size [7], and other advantages, which provide a new process for the production of Al-Cu alloy structural parts. Compared with the traditional subtractive manufacturing process, WAAM has more advantages in the production of large-scale and highly complex Al-Cu alloy structural parts. Geng et al. [8] studied the limit size of products that can be realized by the WAAM process and found that under the thickness of 7.2 mm, the products’ minimum angle and radius of curvature are 20° and 10 mm, respectively, which expands the wield application range of products manufactured by WAAM. Williams et al. [9] found that the wire and arc additive manufacturing process can effectively suppress stress and deformation, which is of great significance to the application of the WAAM process. Cong et al. [10] investigated the effects of different heat sources on the pores and grain structure of Al-Cu alloy deposits and found that under the cold metal transfer (CMT) process, the Al-Cu alloy deposits have fewer pore defects and ideal grain structure. Gu et al. [11] studied the microstructure and mechanical properties of the 2219 alloy manufactured by the CMT process, enhanced the mechanical properties by interlayer rolling and heat treatment, and found that the mechanical properties of vertical direction specimens are worse than those of the horizontal direction specimens, especially elongation. Wang et al. [12] prepared ZL205A alloy by the WAAM process and compared it with the cast sample and found that the structure and properties of the WAAM sample were better than that of the cast sample.

These investigations show that Al-Cu alloy parts can be manufactured by the WAAM process, and that it has a wide range of adaptability on the part structure. The problems at present are the low mechanical properties of the deposit and the anisotropy. The mechanical properties of the WAAM deposit are determined by the microstructure, and the manufacturing process and raw material composition are the key factors to controlling the microstructure. However, the current research mainly focuses on the manufacturing process, and there are few systematic studies on the chemical composition of raw materials.

Cu is the main strengthening element of the Al-Cu alloy, and its function is to precipitate the θ’ phase after solid solution ageing to improve the mechanical properties of the Al-Cu alloy. The content of Cu has an important effect on the physical properties, microstructure, and mechanical properties, which have been studied in casting, forging, and welding processes. For instance, Xing et al. [13] studied the effect of Cu content on the structure and properties of 2219 aluminium alloy forgings and their welding joints and found that reducing the content of Cu can significantly increase the elongation of the alloy. Yu et al. [14] studied the effect of Cu content on the size and distribution of precipitates and found that reducing the content of Cu is beneficial to reducing the aggregation of precipitates and reducing the anisotropy of mechanical properties. Xu et al. [15] found that Cu elements tend to segregate due to the different solidification sequences in large-scale ingots. As a new forming process, WAAM has no obvious reference value for the performance of raw materials in casting, forging, and welding. For example, welding generally considers that the 2024 alloy is not weldable due to hot cracks [16], but it only shows good applicability under the WAAM process [17].

In this paper, deposits with different contents of Cu were prepared by the WAAM process. The contents of Cu are 5.0% (mass fraction, the same as below), 5.65%, and 6.3%; 5.0% is the Cu content of the cast Al-Cu alloy, 6.3% is the deformed Al-Cu alloy Cu content, and 5.65% is the maximum solid solution amount of Cu in Al. The size and distribution of the Al_2_Cu phase were investigated to determine the effect of Cu content on the structure and properties of the deposit. Exploring the appropriate Cu content in Al-Cu alloy raw materials will lay the foundation for the development of Al-Cu alloy wire for the WAAM process.

## 2. Materials and Methods

The raw materials utilized in this research were produced by North East Industrial Materials & Metallurgy Co., Ltd (Fushun, Liaoning province, China). Table 1 lists the chemical composition (mass percentage) of the three kinds of Al-Cu alloy wire with a diameter of 1.2 mm used in this study. The main difference between these three wires is the different contents of Cu.

**Figure 1:**
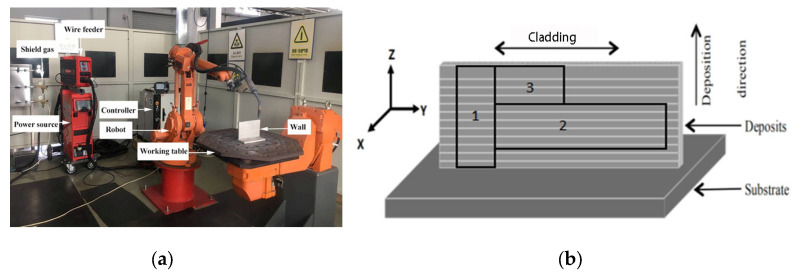
(**a**) The deposition system setup and (**b**) a schematic diagram of the sampling position.

The setup of the WAAM system is shown in Figure 1a, which mainly includes the Fronius TPS4000 power supply and ABB 1410 robot. The sampling position is shown in Figure 1b. The X-axis corresponds to the front of the wall. The Y-axis is the moving direction of the power source, which corresponds to the horizontal direction of the deposit. The Z-axis is the growth direction, which corresponds to the vertical direction of the deposit. The horizontal and vertical tensile samples are taken at positions 1 and 2, respectively. The metallographic and transmission samples are taken at position 3. The tensile sample is processed into a plate shape with a gauge length of 30 mm and a cross-sectional area of 2.5 × 10 mm^2^. 

The samples used for microstructure observation were ground with different types of sandpaper, ground and polished to a mirror surface with 0.5 μm diamond paste, and then etched with mixed acid reagents (1 vol % HF, 1.5 vol % HCl, and 2.5 vol % HNO_3_, the rest is H_2_O). The as-deposit specimen is etched for 20 s, and the specimen after ageing treatment is etched for 25 s. The preparation of transmission electron microscope samples requires the following process: Firstly, manual grinding and polishing is used to prepare thin slices with thicknesses of 60 to 80 microns, and then a disc with a diameter of 3 mm is prepared by a round hole tablet press, and finally the electrolytic double spray is applied; the double spray solution is a methanol solution containing 20% nitric acid, the electrolysis temperature is −25 °C, and the voltage is 16 V. The chemical composition of the wall was measured by OXFORD direct-reading spectrometer, the mechanical properties were measured by WDW-300 microcontrolled electronic universal testing machine, and the pores, cracks, grain size, etc., were observed with a ZEISS Axio observer metallographic microscope (SEM). A Quanta feg250 scanning electron microscope was used to observe the morphology of the precipitated phase and the fracture morphology of the tensile specimen, and the composition of the microarea was analyzed by EDS. The morphologies of nanoscale precipitates were observed by the FEI Titan Themis transmission electron microscope (TEM).

The deposition parameters are shown in Table 2. The heat treatment parameters are as follows: solution temperature is 535 °C, solution time is 360 min, quenching temperature is 40 °C, ageing temperature is 175 °C, ageing time is 240 min, and the quenching medium is water.

## 3. Results and Discussion

### 3.1. Microstructure of the Sample in As-Deposited Status

**Figure 2:**
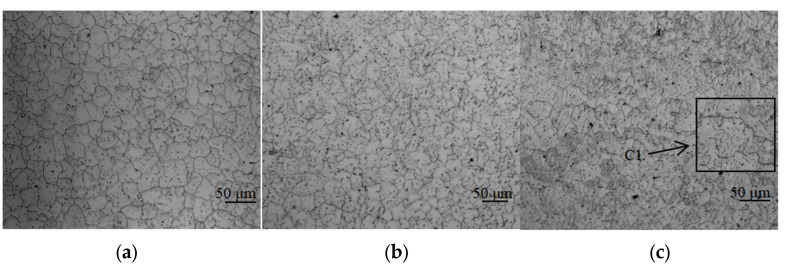
Microstructure of the as-deposited WAAM Al-Cu alloy with different contents of Cu: (**a**) shows the microstructure of the as-deposit with Cu content of 5.0% and the grain size of about 40–50 μm; (**b**)shows the microstructure of the as-deposit with Cu content of 5.65%, and the grain size is about 40–50 μm; (**c**) shows the microstructure of the as-deposit with Cu content of 6.3%, and the grain size is about 40–50 μm, C1 is the region-wide grain boundaries.

The microstructure of the as-deposited walls with different Cu content is shown in Figure 2. When the content of Cu is 5.0%, as shown in Figure 2a, the grain type is uniform equiaxed grains, and the grain size is between 40 μm to 50 μm. This is mainly caused by the fast melting and cooling characteristics of the WAAM process. The solidification speed of the liquid phase metal in the molten pool is fast, the arc has a stirring effect on the molten pool, and the solidification process is dynamic solidification. As the content of Cu increases, the shape and size of the grains do not change, indicating that the content of Cu has no effect on the shape or size of the grains. It can be seen from Figure 2a that when the content of Cu is 5.0%, the precipitated phases are diffusely distributed in the interlayer position and on the grain boundary. As the content of Cu increases to 5.65%, the number of precipitated phases increases significantly, but there is still no segregation phenomenon, as shown in Figure 2b. When the content of Cu continues to increase to 6.3%, the number of precipitated phases continues to increase, as shown in Figure 2c, and the width of the grain boundary is obviously widened, as shown in area C1, which is caused by the segregation of precipitated phases.

**Figure 3:**
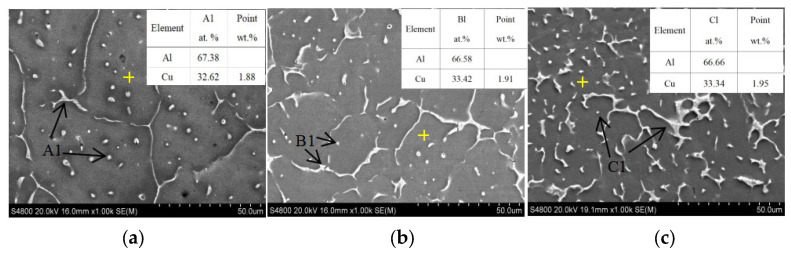
SEM and EDS of the as-deposited WAAM Al-Cu alloy with different contents of Cu: (**a**) is the SEM in the as-deposit with 5.0% Cu content, A1 is θ phase Al_2_Cu, and the content of solid solution Cu in the matrix is 1.88%; (**b**) is the SEM in the as-deposit with 5.65% Cu content, B1 is θ phase Al_2_Cu, and the content of solid solution Cu in the matrix is 1.91%; (**c**) is the SEM in the as-deposit with 6.3% Cu content, A1 is θ phase Al_2_Cu, and the content of solid solution Cu in the matrix is 1.95%.

According to the samples, SEM and EDS pictures with different Cu contents in Figure 3, the main precipitated phase in the grain and on the grain boundary is the θ phase (Al_2_Cu). When the content of Cu is 5.0%, the θ phase appears on the grain boundary as long strips or circular diffuse distribution in the grains, as shown in Figure 3a. As the content of Cu increases to 5.65%, as shown in Figure 3b, the number and size of θ phases increases. When the content of Cu continues to increase to 6.3%, the number of θ phases continues to increase, and precipitates with a size larger than 25 μm × 50 μm appears, as shown in Figure 3c.

At room temperature, the solubility of Cu in Al is 0.05% [18]. Due to the rapid solidification characteristic of the WAAM process, the content of Cu in the deposit has a certain degree of supersaturation, and the Cu contents in the matrices are 1.88%, 1.91%, and 1.95%, respectively. The solid solubility of Cu in as-deposited material is determined by the cooling rate (the cooling rate of these three is under the same process conditions); the solid solubility of Cu in matrix is not much different in as-deposited status, although the Cu content of deposit is different. The remaining Cu in the deposit is precipitated in the form of Al_2_Cu. Therefore, the higher the content of Cu, the more the number of precipitates and the larger the size. The size and distribution of Al_2_Cu have a great influence on the mechanical properties of the alloy [19].

### 3.2. Microstructure of the Deposit after T4 Treatment

**Figure 4:**
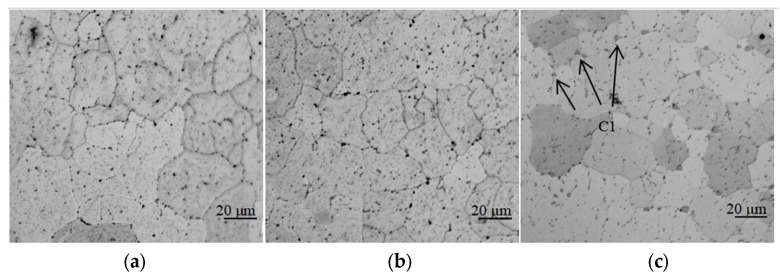
Microstructure of the T4 heat-treated WAAM Al-Cu alloy with different contents of Cu: (**a**) is the microstructure of the accumulated body with 5.0% Cu content under T4 heat treatment, the grain size is 40–50 μm, and no large size precipitated phase is found at the grain boundary; (**b**) is the microstructure of the accumulated body with 5.65% Cu content under T4 heat treatment, the grain size is 40–50 μm, and no large size precipitated phase is found at the grain boundary; (**c**) is the microstructure of the accumulated body with 6.3% Cu content under T4 heat treatment, the grain size is 40–50 μm, and large size precipitated phase is found at the grain boundary (C1).

Figure 4 shows the microstructure of the T4-state WAAM Al-Cu alloy with different Cu contents. The crystal grains of the sample are all equiaxed crystals with uniform size brought by the tempering effect of heat treatment. Due to the genetic effect of the alloy, the grain size of the T4 state is similar to that of the as-deposited state (40 μm to 50 μm). After T4 heat treatment, when the contents of Cu are 5.0% and 5.65%, only the black small-sized precipitates are diffusely distributed in the grain and on the grain boundary, as shown in Figure 4a,b. However, for the 6.3% Cu alloy, after heat treatment, there are not only black small-sized precipitates, but also gray larger-sized precipitates, as shown in Figure 4c (area C1). The size of the gray precipitates is larger than 10 μm, distributed linearly with the grain boundaries, which are parallel to the deposition layers.

**Figure 5:**
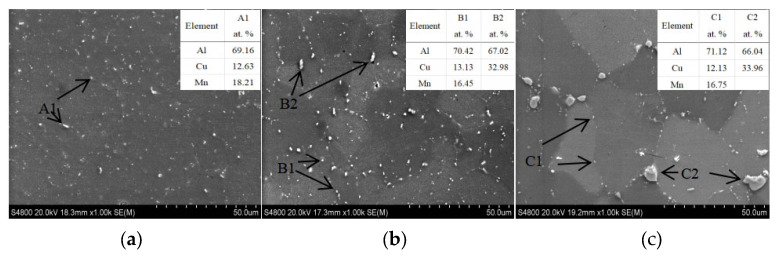
SEM and EDS of the T4 heat-treated WAAM Al-Cu alloy with different contents of Cu: (**a**) is the SEM in the as-deposit with 5.0% Cu content under T4 heat treatment, A1 is T phase; (**b**) is the SEM in the as-deposit with 5.65% Cu content under T4 heat treatment, B1 is T phase, B2 is θ phase; (**c**) is the SEM in the as-deposit with 6.3% Cu content under T4 heat treatment, C1 is T phase, C2 is θ phase.

Figure 5 shows the SEM and EDS images of T4 heat-treated WAAM Al-Cu alloy with different Cu contents. In the sample with a Cu content of 5.0% (Figure 5a), the dispersed phase after heat treatment contains three elements: Al, Cu, and Mn. It is a remelted T phase distributed on the grain boundary, with a size of less than 5 μm, which has pinning effect. Apart from T phases, as shown in Figure 5b area B1, there is also θ phase, as shown in Figure 5b area B2. The size of θ phase is less than 10 μm, and it is diffusely distributed on the grain boundary. 

In the sample with a Cu content of 6.3%, there are still remelted T phases and θ phases, as shown in Figure 5c areas C1 and C2. However, the θ phases are larger in size (greater than 20 μm) and densely distributed. Precipitated phases with this size and distribution on grain boundaries will become the crack initiation positions and affect the mechanical properties of Al-Cu alloy. Compared with the structure of the as-deposited sample, the θ phase after T4 treatment is not completely dissolved into the matrix during the heat treatment process. The higher the Cu content, the larger the size of the precipitated θ phase in the as-deposited sample, and the larger the number and size of the remaining θ phase after solid solution.

**Figure 6:**
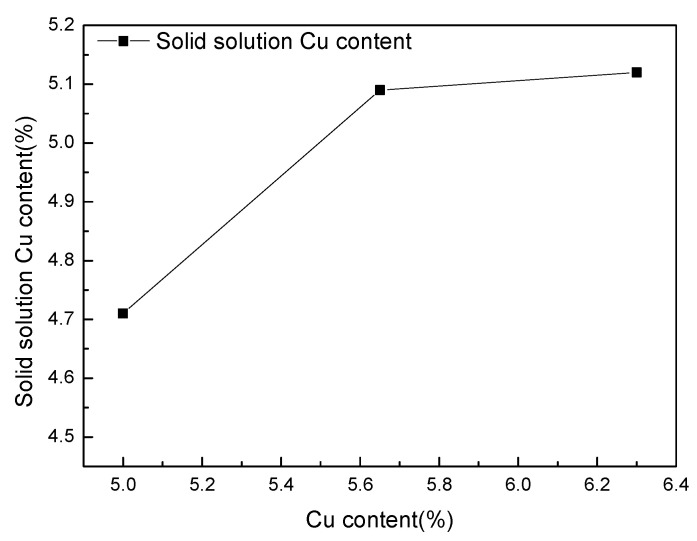
The relationship between the solid-solution Cu content and the content of Cu in the WAAM Al-Cu alloy. When the Cu content is 5.0%, the Cu content in solid solution is 4.71%. When the Cu content is 5.65%, the Cu content in solid solution is 5.09%. When the Cu content is 6.3, the Cu content in solid solution is 5.12%.

The θ phase precipitated in the as-deposited WAAM Al-Cu alloy dissolves into the matrix during the solution treatment to form a supersaturated solid solution, and the θ’ phase precipitates during the ageing process to improve the strength of the alloy. After solution treatment, the content of Cu in the matrix determines the density of the precipitated θ’ phase, thus determining the strength of the alloy. The EDS point measurement of deposit with different contents of Cu under the T4 condition shows that the elements in the matrix are mainly Al, Cu, and Mn, which are also the top three elements in the alloy. Figure 6 proves the Cu content solid solution in the matrix varies with the content of Cu in the WAAM Al-Cu alloy.

When the content of Cu in the deposit is 5.0%, the Cu content dissolved into the matrix after heat treatment is 4.71%, and the other Cu exists in the form of precipitated phase Al_12_Mn_2_Cu. When the Cu content in the deposit increases to 5.65%, the solid solution Cu content also increases, reaching 5.09%, and the remaining Cu exists in the form of the T phase and small-sized θ phase, as shown in Figure 5b. When the Cu content in the deposit is further increased, the solid solution Cu content in the matrix no longer continues to increase, and the remaining Cu is distributed in the grain and on the grain boundary in the form of the T phase and large-size θ phase, as shown in Figure 5c. The amount of solid solution of Cu in the matrix does not increase with the Cu content of the deposit increasing from 5.65% to 6.3%. This is because the solubility of Cu in the Al matrix is controlled by the temperature during the heat treatment process. The limit solid solubility of Cu in Al is 5.29% [20]. According to the Al-Cu alloy phase diagram, the maximum solid solubility of Cu in Al is 5.65% (548 °C). When the content of Cu in the deposit exceeds the limit level, there will be remaining θ phases, and the higher the Cu content, the more remaining large θ phases there will be, which will lose the mechanical properties of the WAAM Al-Cu alloy.

### 3.3. Precipitated Phase under Peak Ageing

**Figure 7:**
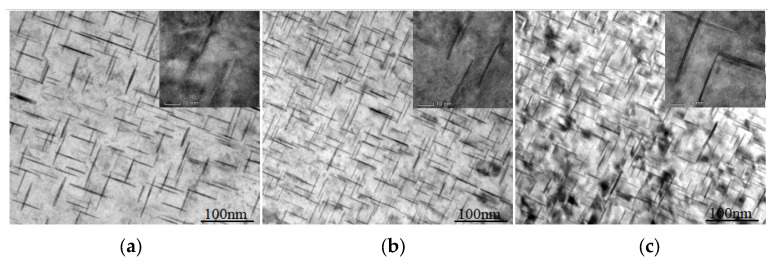
Precipitated phases of WAAM Al-Cu alloys with different Cu contents under peak ageing: (**a**) is the deposit with 5.0% Cu content and T6 heat treatment state; (**b**) is the deposit with 5.65% Cu content and T6 heat treatment state; (**c**) is the deposit with 6.3% Cu content and T6 heat treatment state.

The θ’ phase is the main strengthening phase precipitated during the ageing process of Al-Cu alloys, and the mechanical properties are significantly affected by its size, density, and phase spacing. Figure 7 shows the precipitated phases of the deposit with different content of Cu under peak ageing. The alloy precipitates a platelike phase on the 001-zone axis, and this precipitated phase is the θ’ phase [21]. The three precipitated phases’ sizes are basically the same, approximately 100 nm long, 70 nm wide, and 5 nm thick. When the content of Cu is 5.0%, the distribution of the θ’ phase is relatively sparse, and the phase spacing is about 20 nm. When content of Cu increases to 5.65%, the distribution density of the θ’ phase increases significantly, and the phase spacing is 15 nm. When the content of Cu continues to increase, the distribution density and the phase spacing of the θ’ phase do not change significantly. The number of θ’ phases increases first and then stabilizes under the peak ageing with the increase of the Cu content, which is consistent with the trend of the reaction in Figure 6. The ageing precipitation process of Al-Cu alloy is [22]: supersaturated solid solution (α_ss_) → GP area (GP I area) → θ’’ (GP II area) → θ’ → θ. The quenching process forms vacancy [23]. At the vacancy, Cu atoms are segregated to form the GP region, and then form the θ’ phase. More Cu solid dissolves in the Al matrix, leading to more θ’ phase precipitates.

### 3.4. Mechanical Properties

**Figure 8:**
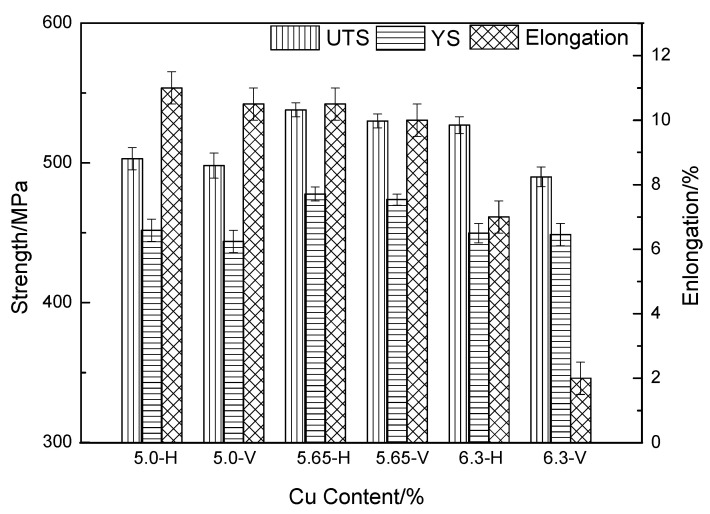
Mechanical properties of WAAM Al-Cu alloys with different Cu contents after T6 heat treatment. 5.0-H and 5.0-V are the horizontal and vertical mechanical properties of the deposit with 5.0% Cu content after T6 heat treatment, respectively; 5.65-H and 5.65-V are the horizontal and vertical mechanical properties of the deposit with 5.65% Cu content after T6 heat treatment, respectively; 6.3-H and 6.3-V are the horizontal and vertical mechanical properties of the deposit with 6.3% Cu content after T6 heat treatment, respectively.

Figure 8 shows the mechanical properties of the WAAM Al-Cu alloy with different Cu contents after T6 heat treatment. H indicates the mechanical properties in the horizontal direction, and V indicates the mechanical properties in the vertical direction (the mechanical property data are the average of many long-term test data). The ultimate tensile strength (UTS), yield strength (YS), and elongation of the Al-5Cu alloy in the horizontal direction are 501 MPa, 452 MPa, and 11%, respectively. The UTS, YS, and elongation of the Al-5Cu alloy in the vertical direction are 498 MPa, 446 MPa, and 10.5%, respectively. When the content of Cu increases to 5.65%, the UTS and YS both increase, reaching 538 MPa and 478 MPa, respectively. When the Cu content increases to 6.3%, the mechanical properties reduce in both horizontal and vertical directions, and the anisotropy of mechanical properties is obvious; particularly, the elongation is only 2% in the vertical direction.

When the content of Cu increases from 5.0% to 5.65%, the Cu content dissolve in the matrix during the solid solution treatment increases, more θ’ strengthening phases can be precipitated, and there is no large-sized remaining θ phase after heat treatment, leading to the improved mechanical properties in both directions. When the Cu content of the deposit increases from 5.65% to 6.3%, a large number of large-size θ phases remain on the grain boundaries as intermetallic compounds after solution treatment, which provides a crack source for the fracture and affects the mechanical properties. It can be observed from Figure 4c that the remaining θ phase is linearly distributed parallel to the deposition layer, so it has a greater impact on the mechanical properties in the vertical direction, resulting in lower mechanical properties in the vertical direction than in the horizontal direction.

### 3.5. Fracture Morphology

**Figure 9:**
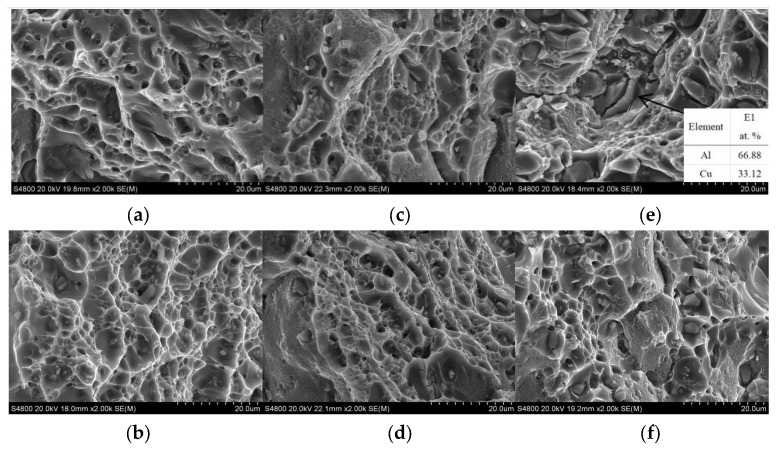
Fracture morphology of samples with different Cu contents: (**a**,**b**) are the vertical and horizontal fracture morphologies of the deposits with 5.0% Cu content after T6 heat treatment; (**c**,**d**) are the vertical and horizontal fracture morphologies of the deposits with 5.65% Cu content after T6 heat treatment; (**e**,**f**) are the vertical and horizontal fracture morphologies of the deposits with 6.3% Cu content after T6 heat treatment.

Figure 9 provides the fracture morphologies of tensile test samples with different Cu contents. For deposits containing 5.0% and 5.65% Cu, the horizontal and vertical fractures are composed of a large number of dimples, showing their good toughness. For the deposits with a Cu content of 6.3%, the vertical fracture is covered by a large number of second-phase particles, with only a small number of dimples, as shown in Figure 9e, indicating that the vertical fracture mode is brittle fracture. EDS energy spectrum shows that these second phases are θ-phase Al_2_Cu, which is dissolved in θ phase after solid solution treatment. It can be observed from Figure 9e that the cracks are first generated on the θ phase, and then propagated to the dimple, resulting in low vertical mechanical properties, especially elongation [24]. The horizontal fracture of deposits with a Cu content of 6.3% is dominated by dimples, indicating that the fracture mode is still ductile fracture.

## 4. Conclusions

In this study, deposits with different Cu contents were prepared by the WAAM process, the microstructure and properties were compared by means of metallography, SEM, EDS, TEM, and mechanical property tests, and the following conclusions were drawn:

- The number and size of θ phases precipitated from the as-deposited Al-Cu alloy increases when the content of Cu increases. When the Cu content is greater than 5.65%, under the solid solution treatment condition, larger remaining θ phases would appear with the higher Cu content.
- After solid solution treatment, the solid solution amount of Cu in the matrix increases first and then remains unchanged with the change of Cu content in the deposit. Under the peak aging state, the number of θ’ phases also shows a trend of first increasing and then remaining unchanged.
- After T6 treatment, the mechanical properties show a trend of first increasing and then decreasing with the increase of Cu content. The mechanical properties achieved the highest level when the content of Cu was 5.65%. The UTS, YS, and elongation reached 538 MPa, 478 MPa, and 10.5%, respectively, which are much higher than those of Al-Cu alloy prepared by the conventional process (UTS is 487 MPa, YS is 428 MPa, and elongation is 5.5%) [12].
- When the content of Cu is greater than 5.65%, the anisotropy of the mechanical properties is obvious, and the vertical fracture mode changes from ductile fracture to brittle fracture.

## Figures and Tables

**Table 1:** Chemical compositions of wires (wt.%).

Alloy	Si	Fe	Cu	Mn	Mg	Cd	Zr	Ti	B	V
Al-Cu5.00	0.040	0.100	5.011	0.421	0.025	0.103	0.177	0.272	0.034	0.125
Al-Cu5.65	0.041	0.102	5.647	0.415	0.026	0.112	0.171	0.278	0.030	0.119
Al-Cu6.30	0.038	0.099	6.304	0.411	0.025	0.120	0.173	0.274	0.032	0.128

**Table 2:** Deposition process parameters.

Process Parameters	
Current	90 A
Voltage	10 V
Travel Speed	8 mm/s
Wire Feed Speed	6.5 mm/min
Gas Flow Rate	25 L/min
Interlayer Cooling Time	120 s

## Data Availability

No new data were created.
