# Correction: Ren et al. The Effect of Cu Content on the Microstructure and Properties of the Wire Arc Additive Manufacturing Al-Cu Alloy. *Materials* 2023, *16*, 2694

**DOI:** 10.3390/ma19132758

**Published:** 2026-06-29

**Authors:** Lingling Ren, Zhenbiao Wang, Shuai Wang, Chengde Li, Wei Wang, Zhu Ming, Yuchun Zhai

**Affiliations:** 1 Inner Mongolia Metal Material Research Institute, Baotou 014000, China15940599525@139.com (C.L.);; 2 North East Industrial Materials & Metallurgy Co., Ltd., Fushun 113000, China; 3 School of Materials and Metallurgy, Northeastern University, Shenyang 110819, China; zhaiyc@mail.neu.edu.cn

Following publication [1], concerns were brought to the attention of the Editorial Office by the Cranfield University regarding the accuracy of the institutional affiliation attributed to co-author Zhenbiao Wang.

In response to the concerns raised, and at the request of the Cranfield University, the affiliation “Welding and Additive Manufacturing Centre, Cranfield University, Bedfordshire MK43 0AL, UK” has been removed. In addition, the contact email address for co-author Zhenbiao Wang has been updated to an alternative correspondence address.

Additionally, the Conflict of Interest statement has also been updated to: Zhenbiao Wang was employed by the company North East Industrial Materials & Metallurgy Co., Ltd. The remaining authors declare that the research was conducted in the absence of any commercial or financial relationships that could be construed as a potential conflict of interest.

The authors state that the scientific conclusions are unaffected. This correction was approved by the Academic Editor. The original publication has also been updated.
